# Impact of miR-146a-3p rs2910164 polymorphism on peri-implantitis susceptibility and target gene-mediated inflammatory responses

**DOI:** 10.2340/aos.v85.45995

**Published:** 2026-05-21

**Authors:** Yajun Zhang, Shaofan Wang, Zeneng Xie, Yinhua Jiang, Tianyi Li

**Affiliations:** a Department of Stomatology, 904th Hospital of Joint Logistic Support Force of PLA, Wuxi, China; b Department of Stomatology, Xiangya Hospital of Central South University, Changsha, China; c School of Stomatology, Zhejiang Chinese Medical University, Hangzhou, China; d Department of Stomatology, The Sixth Affiliated Hospital of Wenzhou Medical University, The People’s Hospital of Lishui, Lishui, China; e Oral and Maxillofacial Surgery, Affiliated Stomatological Hospital of Xuzhou Medical University, Xuzhou, China

**Keywords:** Peri-implantitis, miRNA, SNP, polymorphism, susceptibility

## Abstract

**Objectives:**

This study investigated the association between miR-146a-3p rs2910164 polymorphism and peri-implantitis (PI) susceptibility in the Han Chinese population, as well as the potential mechanisms underlying PI progression.

**Material and Methods:**

183 PI patients and 219 individuals with healthy implants were enrolled. Quantitative polymerase chain reaction was used to detect miR-146a-3p expression levels and analyze the impact of rs2910164 genotypes on these levels. The study compared the association between rs2910164 polymorphism and PI susceptibility, evaluated participants’ periodontal health status, and predicted miR-146a-3p target genes via bioinformatics methods, followed by Gene Ontology (GO), Kyoto Encyclopedia of Genes and Genomes (KEGG), and Protein-Protein Interaction (PPI) enrichment analyses. The regulatory relationship between miR-146a-3p and ITBG7 was validated by dual luciferase assays.

**Results:**

miR-146a-3p expression was elevated in PI patients, with significantly higher levels in those carrying the CG/GG genotypes of rs2910164. Additionally, the GG genotype and G allele of rs2910164 were more prevalent in PI patients, who also exhibited poorer periodontal status. The GG genotype and G allele were identified as risk factors for PI. miR-146a-3p may contribute to PI progression by regulating inflammation-related signaling pathways. The existence of miR-146a-3p and ITBG7 had a negative regulatory relationship.

**Conclusion:**

The miR-146a-3p rs2910164 polymorphism holds promise as a potential biomarker for predicting PI occurrence. miR-146a-3p might modulate inflammation-related signaling pathways via ITBG7 and may play a role in the regulation of PI progression.

## Introduction

Dental implant restoration is a highly effective method for treating oral tooth loss. However, peri-implantitis (PI) remains a key factor leading to implant failure [1]. Recent studies have suggested that PI may exhibit a clustering phenomenon, which could be related to genetic susceptibility [2]. Thus, exploring the association between genetic polymorphisms and PI risk has become a key focus in clinical research. Identifying genetic markers associated with PI risk is of great significance for clinical practice.

MiRNAs play a crucial role in regulating messenger RNAs (mRNAs) [3]. Previous studies have confirmed that abnormal miRNA expression is involved in various oral inflammatory diseases [4]. miR-146a-3p has attracted significant attention in periodontitis research, as it can effectively distinguish between periodontal health and inflammatory states [5]. In experimental models of human periodontal ligament fibroblasts (hPDLFs) under inflammatory conditions, miR-146a-3p has been shown to regulate periodontitis progression [6]. Although periodontitis and PI share similar etiological factors, PI progresses more rapidly [7]. Studies on PI have indicated that miR-146a-3p has potential as a specific and sensitive biomarker for identifying PI patients [8]. Single-nucleotide polymorphisms (SNPs) in miRNAs are important sources of host genetic diversity [9], and related research has become a hot topic.

Numerous studies have explored the association between miR-146a-3p SNPs and susceptibility to cancer and autoimmune diseases [10, 11]. Among these, rs2910164 is one of the most widely studied functional SNPs. For example, in colorectal cancer (CRC), rs2910164 is associated with liver metastasis in Chinese males [12]; in Northeast China, it is linked to an increased risk of lung cancer in non-smoking women [13]; and in a Han Chinese population with oral squamous cell carcinoma, rs2910164 is a risk factor for increased susceptibility [14]. Additionally, rs2910164 is associated with susceptibility to various autoimmune and inflammatory diseases, such as Crohn’s disease (CD) [15] and inflammatory bowel disease (IBD) [16]. Based on the above findings, we hypothesize that miR-146a-3p is abnormally expressed in PI patients, and rs2910164 polymorphism may significantly affect PI progression. However, current research in this area is limited, and the association between PI and rs2910164 polymorphism in the Chinese population remains unstudied.

This study aimed to investigate the effect of miR-146a-3p rs2910164 polymorphism on PI risk and explore the potential mechanisms by which miR-146a-3p regulates PI progression through bioinformatics analysis. The results are expected to provide a new biomarker for PI prediction and a basis for clinical prevention.

## Patients and methods

### Study objects

183 PI patients treated at The Sixth Affiliated Hospital of Wenzhou Medical University and The People’s Hospital of Lishui between May 2020 and September 2023 were included in the case group, and 219 individuals with healthy implants were selected as the control group.

The selection of PI patients for this study was strictly based on the clinical diagnostic guidelines for PI established by the International Team for Implantology (ITI). Additionally, the diagnostic criteria were developed in alignment with the clinical diagnosis and treatment standards of Periodontology. To ensure objectivity, standardization, and accuracy, the diagnosis for all study participants was independently conducted by two periodontal specialists. Inclusion criteria for PI patients: (a) Implants in place for at least 3 months; (b) No bite trauma to the implant; (c) Presence of bleeding on probing, suppuration, or elevated blood concentration; (d) Probing depth (PD) ≥ 6 mm; (e) Radiographic evidence of peri-implant bone loss > 2.5 mm, measured via cone-beam computed tomography (CBCT) at the site defined as the vertical distance from the implant shoulder to the apical extent of osseointegration. Intraclass correlation coefficients (ICCs) were calculated to evaluate inter- and intra-examiner reliability of peri-implant bone resorption measurements, with assessments conducted across multiple time points to mitigate measurement variability arising from examiner differences or temporal inconsistency.

Control group criteria: No signs of gingival inflammation (e.g. soft tissue redness or bleeding), no increased PD, and no radiographic evidence of peri-implant bone loss assessed by the same CBCT measurement protocol as the case group.

Exclusion criteria: (1) Undergoing orthodontic treatment; (2) Received immunotherapy, antibiotics, or similar treatments within the past 3 months; (3) Suffering from systemic diseases or acute/chronic infections; (4) Presence of other local lesions (e.g. oral infections, jaw cysts); (5) Pregnant or lactating women; (6) Inflammation caused by iatrogenic factors; (7) Incomplete clinical data.

All participants provided informed consent. Clinical baseline information, including age, gender, smoking/drinking history, periodontitis history, and oral hygiene habits, was collected. Periodontal status indicators of PI patients were extracted from clinical periodontal assessment reports.

### Serum collection

After enrollment, 10 mL of blood was collected from each participant. All peripheral venous blood samples from the study participants were taken in the early morning while fasting (before 10 a.m.), with strict control over the collection time window to minimize experimental errors due to variations in serum composition at different times. Additionally, the blood collection was performed by trained nurses following standardized procedures and sterile techniques. The sample was centrifuged at 4°C and 3,000 r/min for 15 min, and the supernatant serum was transferred to a 2 mL RNase-free EP tube.

### DNA and RNA extraction

Genomic DNA was extracted from serum samples using the MolPure® Mag96 Blood DNA Kit (Yeasen, China). The serum sample was treated with protein lysis buffer and incubated at room temperature (25°C) for 20 min. The entire process was vortexed and shaken three times to ensure complete lysis. The magnetic bead binding step was performed at room temperature (25°C) for 15 min, with gentle mixing. Washing and elution were strictly carried out according to the kit instructions. The DNA extraction yield per 10 ml serum sample was 1.2–2.5 μg, and the extraction efficiency remained consistent, meeting the requirements for sample size and purity for subsequent detection. DNA purity was assessed using a NanoDrop ultra-micro spectrophotometer, showing OD260/280 values between 1.8 and 2.0, and OD260/230 values between 2.0 and 2.2, indicating the absence of protein, phenol, and other contaminants.

Total RNA was extracted from serum samples using the SteadyPure Blood Serum and Plasma RNA Extraction Kit (Accurate, China), and RNA integrity was evaluated with an Agilent 2100 Bioanalyzer, ensuring RIN values of 7.0 or higher. This guarantees effective cDNA synthesis and reliable results of gene expression detection. The cDNA was synthesized with the BeyRT™ III cDNA First Strand Synthesis Kit (Beyotime, China). A 20 μL reaction system was prepared using 1 μg of total RNA as the template, which included 4 μL of 5× RNA Reaction Buffer, 1 μL of 20 U/μL RNase Inhibitor, 2 μL of 10 mM dNTP Mix, 1 μL of Oligo(dT)18 Primer, and 1 μL of BeyoRT™ III M-MLV reverse transcriptase, with DEPC-treated water added to reach the final volume. The reaction mixture was first incubated at 65°C for 5 min in an ice bath to denature RNA secondary structures. It was then incubated at 42°C for 15 min to complete the reverse transcription. Finally, the reaction was stopped by heating at 85°C for 5 s, and the resulting product was stored at −20°C for future use. All experimental conditions, including reaction components, temperatures, and durations, were strictly controlled.

### Quantitative polymerase chain reaction

The miRNA Unimodal SYBR quantitative polymerase chain reaction (qPCR) Master Mix (Vazyme, China) was used to detect the relative expression of miR-146a-3p in serum. miR-146a-3p and ITGB7-specific primers were synthesized by Genscript Biotechnology Co., Ltd. (Nanjing, China), with sequences (5’-3’): forward primer TGGAACTGAATTCCATGGTT and reverse primer CTGAAGACTGAATTTCAGAGG. Cel-miR-39 was used as the internal reference gene, with the primer sequence (5’-3’) UCACCGGGUAAAUCAGCUUG.

According to the manufacturer’s protocol, a 20 μL qPCR reaction system was prepared; the following components were required: 10 μL of 2 × miRNA Unimodal SYBR qPCR Master Mix, 0.4 μL of Forward Primer (10 μM), 0.4 μL of Reverse Primer (10 μM), 1 μL of Template cDNA, and 8.2 μL of double-distilled water. Detection was performed using a Mastercycler EP RealPlex real-time fluorescence quantitative PCR instrument (Eppendorf AG, Germany), under the following conditions: initial denaturation at 95°C for 30 s, followed by 40 cycles consisting of denaturation at 95°C for 10 s and annealing/extension at 60°C for 30 s. Dissolution curves were generated using the instrument’s default settings. All qPCR experiments were carried out strictly following MIQE guidelines, with multiple replicates included throughout to ensure reproducibility. The procedures were standardized and performed by trained personnel.

### rs2910164 polymorphism detection

The rs2910164 polymorphism was detected using the Hieff Unicon® qPCR TaqMan Probe Master Mix (Yeasen, China). The primer sequences (5’-3’) for rs2910164 were: forward primer CATGGGTTGTCAGTCAGAGCT and reverse primer TGCCTTCTGTCTCCAGTCTTCCAA.

A 20 μL PCR amplification system was prepared. The reaction plate was placed in a Mastercycler EP RealPlex real-time fluorescence quantitative PCR instrument for amplification. After the reaction, genotypes were determined by analyzing the peak shape and signal intensity of the amplification curve, supplemented by manual verification to ensure accuracy.

### Functional analysis

miRNA target genes were predicted using TargetScan, MiRDB, and miRWalk databases. The intersection of predicted target genes from these databases was used as the gene set for subsequent analysis. The criteria for selecting overlapping genes were: Target Score > 80 in MiRDB and binding *P*-value > 0.8 in miRWalk.

Target genes were analyzed using the DAVID database. GO annotations with *P* < 0.05 were considered significant. KEGG pathway enrichment analysis was performed on predicted target genes.

Utilizing STRING, identified protein products that exhibited interactions with one another and constructed a diagram representing the protein interaction network.

### Luciferase report assay

Human primary periodontal ligament fibroblasts (hPDLFs) were procured from Hefei Wanwu Biotechnology Co., Ltd. in China. These cells were cultured in an environment at 37°C with 5% CO_2_, utilizing the matched complete culture medium. The wild-type (wt) and mutant (mut) sequences of ITGB7, which contain binding sites for miR-146a-3p, were cloned into the pmiR GLO dual luciferase reporter vector to generate the recombinant plasmids ITGB7-wt and ITGB7-mut. Site-directed mutagenesis via PCR was used to create the ITGB7-mut plasmid by introducing nucleotide substitutions at the predicted binding site, disrupting its interaction with miR-146a-3p. Sanger sequencing confirmed the correct mutation and no unintended changes. Subsequently, these vectors were co-transfected into hPDLFs, which were in the logarithmic growth phase, alongside miR-146a-3p mimetics (miR-146a-3p mimic) and a negative control (miR-146a-3p mimic NC) using Lipofectamine 3000 (Thermo, USA) as the transfection reagent. Following a 48-h incubation period, luciferase activity was assessed in the cells in accordance with the manufacturer’s instructions (Beyotime, China).

### Statistical analysis

Measurement data were expressed as mean ± SD, and categorical data as counts and percentages (%). Intergroup comparisons were performed using t-tests, one-way analysis of variance, or the Mann-Whitney U test as appropriate; categorical data were compared using chi-square tests. Odds ratios (OR) and 95% confidence intervals (CI) were calculated to assess correlation strength. Hardy-Weinberg equilibrium (HWE) was used to evaluate population genetic equilibrium. Utilized receiver operating characteristic (ROC) curves to evaluate the diagnostic effectiveness of miR-146a-3p in PI patients. Utilized Pearson correlation analysis to examine the relationship between serum miR-146a-3p levels and ITGB7 expression in PI patients.

## Results

### Demographic and clinical information

There were no significant differences in age (*P* = 0.586) or gender ratio (*P* = 0.281) between the PI case group and the control group, indicating good comparability (Table 1). Lifestyle factors (smoking, drinking), daily oral hygiene habits (brushing, flossing, rinsing), reasons for tooth loss, implant location, and peri-implant phenotype also showed no significant differences between the two groups (*P* > 0.05, Table 1).

**Table 1:** Clinical information of all subjects.

Characteristics	Healthy Implants (*N* = 219)	Peri-Implantitis (*N* = 183)	*P*
Age (years) (mean ± SD)	43.57 ± 5.28	43.28 ± 5.24	0.586
Gender (*n*/%)			0.281
Male	129 / 58.90	98 / 53.55	
Female	90/41.10	85/46.45	
Drinking history (*n*/%)			0.841
Yes	65 / 29.68	56 / 30.60	
No	154 / 70.32	127 / 69.40	
Smoking history (*n*/%)			0.896
Yes	80 / 36.53	68 / 37.16	
No	139 / 63.47	115 / 62.84	
Periodontitis history (*n*/%)			0.024
Yes	89 / 40.64	95 / 51.91	
No	130 / 59.36	88 / 48.09	
Tooth loss reason (*n*/%)			0.658
Periodontitis	110 / 50.23	94 / 51.37	
Deep caries	5 / 2.28	2 / 1.09	
Trauma	104 / 47.49	87 / 47.54	
Position (*n*/%)			0.055
Anterior region	73 / 33.33	78 / 42.62	
Posterior region	146 / 66.67	105 / 57.38	
Per-implant phenotype (*n*/%)			0.252
Thin	94 / 42.92	89 / 48.63	
Thick	125 / 57.08	94 / 51.37	
Brushing daily (times) (*n*/%)			0.493
1–3	185 / 84.47	159 / 86.89	
> 3	34 / 15.53	24 / 13.11	
Dental floss daily (*n*/%)			0.065
Yes	82 / 37.44	76 / 41.53	
No	44 / 20.09	21 / 11.48	
Infrequent	93 / 42.47	86 / 46.99	
Mouth washing daily (*n*/%)			0.419
Yes	66 / 30.13	66 / 36.07	
No	45 / 20.55	37 / 20.22	
Infrequent	108 / 49.32	80 / 43.71	
Gingival index (mean ± SD)	0.66 ± 0.58	2.09 ± 0.70	< 0.001
Plaque index (mean ± SD)	0.86 ± 0.52	2.29 ± 0.80	< 0.001
Calculus index (mean ± SD)	0.32 ± 0.24	0.70 ± 0.47	< 0.001
PPD (mm) (mean ± SD)	1.90 ± 0.54	5.30 ± 0.96	< 0.001
CAL (mm) (mean ± SD)	1.60 ± 0.62	4.69 ± 1.32	< 0.001

SD: standard deviation; PPD: peri-implant pocket depth; CAL: clinical attachment level.

*P* < 0.05 means a significant difference.

However, significant differences were observed in periodontitis history (*P* = 0.024) and periodontal status indicators (*P* < 0.001). The PI case group had significantly higher levels of these indicators, indicating poorer periodontal status (Table 1).

### Relative expression levels of miR-146a-3p

**Figure 1:**
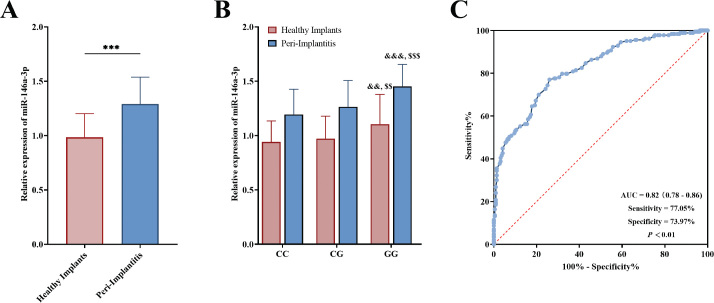
Serum miR-146a-3p levels in PI patients and controls detected by qPCR (A), and miR-146a-3p levels in different rs2910164 genotypes (CC, CG, GG) analyzed by qPCR (B), as well as the diagnostic value of miR-146a-3p in PI patients via ROC curve analysis (C). ***, *P* < 0.001 versus healthy implants. &&, *P* < 0.01; &&&, *P* < 0.001 versus CC genotype. $$$, *P* < 0.001 versus CG genotype.

Serum miR-146a-3p expression was significantly higher in the PI case group (*P* < 0.001, Figure 1A).

In the PI case group, there were 44 cases with the CC genotype, 98 with CG, and 41 with GG; in the control group, 74 with CC, 110 with CG, and 35 with GG (*P* = 0.06). miR-146a-3p expression was significantly higher in both CG and GG genotypes (*P* < 0.01, Figure 1B). Additionally, miR-146a-3p expression in PI patients with the GG genotype was significantly higher than in those with the CG genotype (*P* < 0.001, Figure 1B). The results of the ROC analysis indicated that the area under the curve (AUC) for serum miR-146a-3p in the diagnosis of PI was 0.82 (95% CI: 0.78–0.86). The sensitivity and specificity of miR-146a-3p were found to be 77.05% and 73.97%, respectively. These findings suggested that serum miR-146a-3p demonstrated a significant capacity for identifying PI (*P* < 0.01, Figure 1C).

### Association between miR-146a-3p rs2910164 polymorphism and PI susceptibility

Genotype distributions in both the control group and PI case group were in Hardy-Weinberg equilibrium (*P*_HWE_ values of 0.579 and 0.335, respectively).

The frequency of the CC genotype of miR-146a-3p rs2910164 was lower in the PI case group (24.04%) than in the control group (33.79%), while the frequency of the GG genotype was higher in the PI case group (22.41%) than in the control group (15.98%). The CG genotype frequency showed a slight difference (53.55% in the PI group vs. 50.23% in the control group, Table 2).

**Table 2:** Association between miR-146a-3p rs2910164 polymorphism and peri-implantitis susceptibility.

Genotype/Allele	Healthy implants (*N* = 219)	Peri-Implantitis (*N* = 183)	*P*	OR (95% CI)
CC	74 (33.79)	44 (24.04)	-	-
CG	110 (50.23)	98 (53.55)	0.085	1.498 (0.944–2.378)
GG	35 (15.98)	41 (22.41)	0.022	1.970 (1.097–3.538)
C	258 (58.90)	186 (50.82)	-	-
G	180 (41.10)	180 (49.18)	0.022	1.387 (1.049–1.835)
*P*_HWE_	0.579			

OR: odds ratio; 95% CI: 95% confidence interval; *P*_HWE_: *P*-value for Hardy-Weinberg equilibrium test.

*P* < 0.05 means a significant difference.

Notably, the GG genotype was more prevalent in the PI case group (*P* = 0.022), and carriers of the GG genotype had a 1.970-fold increased risk of PI (95% CI: 1.097–3.538, Table 2).

The frequency of the G allele was significantly higher in the PI case group (49.18%) than in the control group (41.10%, *P* = 0.022, Table 2). Carriers of the G allele had a 1.387-fold increased risk of PI (95% CI: 1.049–1.835, Table 2).

### Periodontal status of PI patients with different miR-146a-3p rs2910164 genotypes

PI patients were divided into three groups based on rs2910164 genotypes (CC, CG, GG), and their periodontal status indicators were compared. Patients with the CC genotype had significantly lower levels of gingival inflammation, plaque accumulation, calculus index, peri-implant pocket depth (PPD), and clinical attachment level (CAL); in contrast, those with the CG or GG genotype had higher levels of these indicators (*P* < 0.001, Table 3), indicating poorer periodontal status in CG or GG genotype carriers.

**Table 3:** Periodontal status in peri-implantitis patients with miR-146a-3p rs2910164 polymorphism.

Parameters	CC (*n* = 44)	CG (*n* = 98)	GG (*n* = 41)	*P*
Gingival index	1.75 ± 0.62	1.97 ± 0.62	2.73 ± 0.55	< 0.001
Plaque index	2.25 ± 0.68	2.28 ± 0.82	2.35 ± 0.89	< 0.001
Calculus index	0.69 ± 0.41	0.70 ± 0.48	0.71 ± 0.52	< 0.001
PPD (mm)	5.10 ± 0.88	5.26 ± 0.91	5.63 ± 1.06	< 0.001
CAL (mm)	4.54 ± 1.10	4.74 ± 1.42	4.74 ± 1.33	< 0.001

PPD: peri-implant pocket depth; CAL: clinical attachment level.

*P* < 0.05 means a significant difference.

### Function and target gene prediction of miR-146a-3p

**Figure 2:**
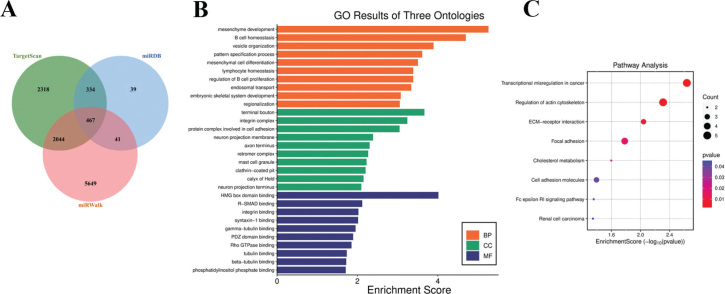
Venn diagram of predicted downstream target genes of miR-146a-3p predicted by TargetScan, MiRDB and miRWalk databases (A), and GO (B) and KEGG (C) enrichment analyses of target genes via DAVID database.

To explore the role and potential regulatory mechanisms of miR-146a-3p in PI onset and progression, downstream target genes of miR-146a-3p were analyzed. TargetScan predicted 5,163 targets, miRDB 881, and miRWalk 8,201. Cross-referencing these databases identified 467 candidate target genes (Figure 2A).

Enrichment analysis of candidate target genes yielded 30 enriched GO terms and eight potential KEGG pathways, with all detailed information on significantly enriched entries and pathways summarized in Supply Table 1. This table comprehensively presents the ID, name, gene ratio, exact *P*-value, associated gene IDs, and the number of enriched genes for each GO annotation term and KEGG signaling pathway, providing a complete basis for assessing the functional distribution and regulatory pathways of miR-146a-3p target genes.

In the biological process (BP) category, enrichment was observed in mesenchyme development, B cell homeostasis, etc. (Figure 2B). In the cellular component (CC) category, significant enrichment was found in terminal bouton, integrin complex, etc. (Figure 2B). In the molecular function (MF) category, enrichment was observed in HMG box domain binding, R-SMAD binding, etc. (Figure 2B).

KEGG pathway enrichment analysis identified eight signaling pathways, including transcriptional mis-regulation in cancer and regulation of actin cytoskeleton (Figure 2C).

**Figure 3:**
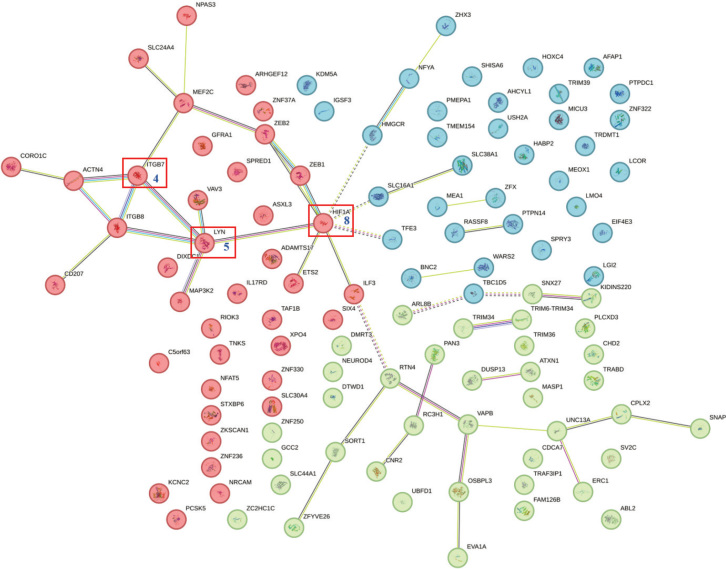
Protein interaction network of miR-146a-3p target gene constructed by bioinformatics analysis.

A protein-protein interaction (PPI) network of potential target genes was established utilizing the STRING database to investigate their interactions. The analysis of the PPI network revealed the three most central genes, namely HIF1A, LYN, and ITGB7, which exhibited degree values of 8, 5, and 4, respectively (Figure 3).

### Relationship between miR-146a-3p and ITGB7

Previous research has indicated that HIF1A and LYN exhibit up-regulation in patients with PI. Given the consistent expression levels of these genes in relation to miR-146a-3p, it is unlikely that a direct regulatory relationship exists between them. Consequently, further investigations will focus on ITGB7 as a potential target gene.

**Figure 4:**
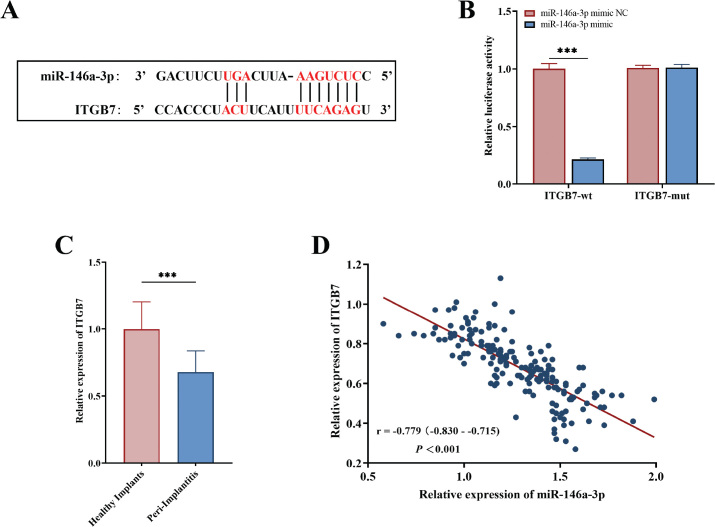
The binding sites between miR-146a-3p and *ITBG7* by bioinformatics tools (A), as well as the dual luciferase activity of *ITBG7*-wt and *ITBG7*-mut regulated by miR-146a-3p in hPDLFs cells (B). The serum levels of *ITBG7* in the PI patients and controls detected by quantitative assay (C), and the correlation between miR-146a-3p and *ITBG7* via Pearson correlation analysis (D). ***, *P* < 0.001 versus healthy implants.

Predictive analyses from databases identified binding sites between miR-146a-3p and ITGB7 (Figure 4A). Subsequent dual luciferase assays demonstrated that the overexpression of miR-146a-3p significantly reduced the luciferase activity of the wild-type ITGB7 (*P* < 0.001), while no significant effect was observed on the mutant variant (*P* > 0.05, Figure 4B).

qPCR analysis revealed that the expression level of ITGB7 in the serum of patients within the PI case group was markedly lower than that of the control group (*P* < 0.001, Figure 4C). Additionally, Pearson correlation analysis indicated a significant negative correlation between miR-146a-3p and ITGB7 (*r* = −0.779, *P* < 0.001, Figure 4D).

## Discussion

PI is a major cause of dental implant failure. Recent systematic reviews have shown a gradual increase in PI prevalence, particularly at implant sites, suggesting that genetic factors may play an important role in its onset [17]. Therefore, clarifying the risk factors and mechanisms of PI is crucial for preventing peri-implant infections.

Previous studies have confirmed that miRNAs are involved in the pathogenesis of inflammatory diseases [18]. miR-146a-3p regulates the transcription factor NF-κB during inflammation [6] and acts as a key signaling molecule targeting TLR and IL-1 pathways [19], playing an important role in regulating pro-inflammatory cytokine production and macrophage polarization [20]. Studies have shown that miR-146a-3p is upregulated in the GCF of periodontitis patients [5] and in PI tissues [8], and its high expression is associated with severe inflammatory responses, suggesting its potential as a biomarker for identifying affected populations. Over 80% of GCF components are derived from serum, indicating high similarity between the two. Using serum as a sample ensures an adequate sample size [21]. In contrast, oral tissue samples are limited in quantity and prone to contamination by bacteria and mycoplasma, making RNA extraction difficult [22]. Therefore, serum was chosen as the sample for analysis in this study. Consistent with previous findings, this study found that the periodontal health of PI patients was found to be compromised, and similarly elevated levels of miR-146a-3p were observed in their serum, as well as miR-146a-3p exhibited a significant capacity to differentiate PI patients, indicating a possible link between miR-146a-3p and the development of PI. Considering the different sizes and types of implants, as well as different surgical implantation methods, may affect the local inflammatory microenvironment and potentially impact the expression of miRNAs and the risk of PI occurrence. To address this issue, subsequent research will further refine and report on the baseline distribution of implant types, sizes, and implantation methods of the enrolled subjects, and explore their potential impacts in depth to enhance the scientific rigor and universality of the research conclusions.

In this study, an HWE test was performed on the genotype distribution of rs2910164 to confirm the genetic equilibrium of the study population. This ensures that the research subjects represent randomly selected populations without significant bias factors such as population migration, natural selection, or consanguineous marriage. It also verifies that the genotype frequency distribution aligns with the principles of population genetics, providing a genetic foundation for the reliability of subsequent association analyses. Additionally, it is important to note that deviations from HWE may result from factors such as sample selection bias, population stratification, natural selection pressures at gene loci, experimental errors, or underlying demographic differences within the study population. SNPs in gene regions can affect gene expression and maturation, leading to abnormal gene regulation [23]. Numerous studies have explored the association between SNPs and PI risk, mainly focusing on inflammatory cytokine genes. For example, a study in the Portuguese population found that the mutant alleles of IL-1A (-889) and IL-1B (+3954) were more common in PI patients, associated with increased PI susceptibility [24]. A meta-analysis of Asian populations showed a strong association between TNF-α (-308 G > A) and PI, with its upregulation increasing pro-inflammatory factors and stimulating osteoclast gene activity [25]. miRNAs have become a focus of clinical research due to their stability, sensitivity, specificity, and ease of detection. As mentioned earlier, rs2910164 is one of the most widely studied functional SNPs in miR-146a-3p, and its polymorphism is associated with increased risk of CRC (G > C), lung cancer (GG > CG), oral squamous cell carcinoma (GG > CG/CC), and other cancers in the Chinese population [12–14]. A study in the Iranian population found that individuals carrying the CG/GG genotype of rs2910164 had an increased risk of PI [26], consistent with the findings of this study. This study found that the rs2910164 GG genotype and G allele were more common in PI patients, who also had poorer periodontal health, higher serum miR-146a-3p levels, and increased disease risk compared with C allele carriers. These results suggest that miR-146a-3p rs2910164 polymorphism is an important factor affecting PI susceptibility, and G allele carriers have a higher PI risk after adjusting for age, gender, lifestyle, diabetes, and iatrogenic factors.

In recent years, miR-146a-3p has become a research focus, with numerous studies highlighting its association with tumors and inflammation [27]. However, its specific role and regulatory mechanisms in PI remain unclear. This study’s enrichment analysis showed that miR-146a-3p target genes can bind to protein domains, which are essential for protein translation and function [28]. These target genes play an important role in regulating immune cell homeostasis and proliferation, as well as stromal cell development and differentiation, which are involved in various inflammatory tissue injuries [29]. Additionally, the eight KEGG pathways identified are related to tumorigenesis and inflammation and are involved in regulating TLR signaling, NF-κB transcription factors, and cytokines (e.g. IL, TNF) in the body [30]. Polymorphisms or mutations outside the pre-miRNA neck ring structure and miRNA seed region can affect miRNA processing and its interaction with target genes, reducing targeting efficiency and causing variability in regulatory pathways [31]. These findings suggest that different rs2910164 genotypes may affect the structural stability and function of the miR-146a-3p gene in PI patients, thereby regulating the body’s inflammatory response and contributing to PI onset and progression.

The PPI network analysis has identified three principal core genes: HIF1A, LYN, and ITGB7. Notably, HIF1A serves as a biomarker for assessing tissue hypoxia, which is associated with the prolonged effects of mastication and the presence of bacteria, particularly anaerobic bacteria. Research indicates that the expression of HIF1A is significantly elevated in the tissues of patients with PI [32]. LYN is recognized as a critical target in the context of periodontitis, with its upregulation contributing to the mitigation of inflammatory responses [33]. ITGB7, predominantly expressed in leukocytes, plays a role in the body’s inflammatory processes [34]. In this study, we observed a downregulation of ITGB7 levels in the serum of PI patients, which appears to have a negative regulatory relationship with miR-146a-3p. Consequently, we hypothesized that the rs2910164 might influence the expression of ITGB7 by modulating the levels of miR-146a, thereby participating in the inflammatory response in patients with PI.

This study has certain limitations. First, the functional role of miR-146a-3p in PI awaits further validation through in vitro and in vivo experiments. Second, our initial study cohort was restricted to a single-center Han Chinese population, a selection made to minimize ethnic genetic confounding and enhance the robustness of the primary genetic association analysis, yet this homogeneous sample inevitably limits the generalizability of our findings to other ethnic groups or geographically distinct populations, and future research will expand to multicenter, multi-ethnic cohorts including ethnic minorities and regionally diverse Han subgroups to validate the current findings and further assess the effects of the rs2910164 polymorphism across different genetic backgrounds. Third, the present study only utilized serum samples for detection, and subsequent analyses will simultaneously measure and compare miR-146a-3p expression levels in serum, gingival crevicular fluid (GCF), and gingival tissue from PI patients to distinguish its systemic and local expression characteristics and clarify its association with local pathological changes. Fourth, the tissue origin of serum miR-146a-3p and its specific correlation with peri-implant local inflammation remain unclear, and to address this critical issue, we will perform a series of targeted studies, including measuring miR-146a-3p levels in GCF, gingival tissue, peripheral blood mononuclear cells and serum of PI patients, localizing its expression in lesion tissues via in situ hybridization to identify its primary tissue source, conducting in-depth correlation analysis between serum miR-146a-3p levels and both clinical local inflammation indicators (such as PD and attachment loss) and molecular markers (such as GCF inflammatory factors), and verifying whether the local inflammatory microenvironment drives the release of miR-146a-3p into the systemic circulation using in vitro cell models and in vivo animal models. In addition, we will conduct a 3–5-year long-term follow-up of all enrolled participants, systematically tracking key indicators including PI onset time, PD and peri-implant bone resorption, collecting detailed genotype-stratified clinical treatment plans and therapeutic outcomes (such as inflammation resolution and bone tissue repair), and applying survival analysis and stratified modeling to comprehensively evaluate the association of the rs2910164 polymorphism with PI onset risk, disease progression rate and treatment response, thereby clarifying its clinical utility as a prognostic biomarker for PI and providing more robust evidence for the personalized prognosis assessment and precision treatment of PI patients.

## Conclusions

In summary, this study found that miR-146a-3p expression is significantly elevated in PI patients. The rs2910164 GG genotype and G allele are potential risk factors for PI. miR-146a-3p may be involved in PI progression by regulating inflammation-related signaling pathways via ITBG7. These findings highlight the potential of rs2910164 as a predictive marker for PI susceptibility, providing important insights for the early identification and personalized clinical management of PI patients.

## Supplementary Material


