# Supplementary material for: Impact of miR-146a-3p rs2910164 polymorphism on peri-implantitis susceptibility and target gene-mediated inflammatory responses
Source: Acta Odontol Scand. 2026 May 21;85:45995. doi: 10.2340/aos.v85.45995 (PMC13200258; doi:10.2340/aos.v85.45995)
Supplement: Supplementary file 1 [file AOS-85-45995-s1.pdf]

Supplementary material has been published as submitted. It has not been copyedited or typeset by Acta Odontologica Scandinavica.

Supply table 1 Significantly enriched GO and KEGG terms, along with their gene counts and P values

ID	Description	GeneRatio	pvalue	geneID	Count
GO:0060485	mesenchyme development	10/106	5.47738E-06	BNC2/SPRED1/RTN4/HIF1A/CORO1C/ZEB2/SIX4/IL17RD/MEF2C/MEOX1	10
GO:0001782	B cell homeostasis	4/106	2.00665E-05	RC3H1/HIF1A/MEF2C/LYN	4
GO:0016050	vesicle organization	9/106	0.000127755	SNAP91/UNC13A/STXBP6/CORO1C/ZEB2/VAPB/CPLX2/SORT1/VAV3	9
GO:0007389	pattern specification process	10/106	0.000243642	HOXC4/HIF1A/PCSK5/ZEB2/TRAF3IP1/MEF2C/DMRT3/ZEB1/ETS2/MEOX1	10
GO:0048762	mesenchymal cell differentiation	7/106	0.000311822	SPRED1/RTN4/HIF1A/CORO1C/ZEB2/IL17RD/MEF2C	7
GO:0002260	lymphocyte homeostasis	4/106	0.000408817	RC3H1/HIF1A/MEF2C/LYN	4
GO:0030888	regulation of B cell proliferation	4/106	0.000408817	RC3H1/MEF2C/LYN/VAV3	4
GO:0016197	endosomal transport	7/106	0.000456596	SNX27/TBC1D5/ERC1/GCC2/CORO1C/ARL8B/SORT1	7
GO:0048706	embryonic skeletal system development	5/106	0.000835747	HOXC4/PCSK5/SIX4/MEF2C/ZEB1	5
GO:0003002	regionalization	8/106	0.000883602	HOXC4/PCSK5/ZEB2/TRAF3IP1/MEF2C/DMRT3/ETS2/MEOX1	8
GO:0043195	terminal bouton	4/103	0.000215858	KCNC2/UNC13A/CPLX2/USH2A	4
GO:0008305	integrin complex	3/103	0.0005727	ITGB8/LYN/ITGB7	3

GO:0098636	protein complex involved in cell adhesion	3/103	0.000892497	ITGB8/LYN/ITGB7	3
GO:0032589	neuron projection membrane	3/103	0.004090612	KCNC2/SHISA6/USH2A	3
GO:0043679	axon terminus	4/103	0.004961751	KCNC2/UNC13A/CPLX2/USH2A	4
GO:0030904	retromer complex	2/103	0.005402919	SNX27/TBC1D5	2
GO:0042629	mast cell granule	2/103	0.005922903	CPLX2/LYN	2
GO:0005905	clathrin-coated pit	3/103	0.006254708	TBC1D5/SNAP91/SORT1	3
GO:0044305	calyx of Held	2/103	0.007028466	UNC13A/CPLX2	2
GO:0044306	neuron projection terminus	4/103	0.007986384	KCNC2/UNC13A/CPLX2/USH2A	4
GO:0071837	HMG box domain binding	3/105	9.65416E-05	HOXC4/MEF2C/MEOX1	3
GO:0070412	R-SMAD binding	2/105	0.007585	PMEPA1/ZEB2	2
GO:0005178	integrin binding	4/105	0.009429827	ACTN4/ITGB8/LYN/ITGB7	4
GO:0017075	syntaxin-1 binding	2/105	0.009635638	UNC13A/CPLX2	2
GO:0043015	gamma-tubulin binding	2/105	0.01112416	DIXDC1/LYN	2
GO:0030165	PDZ domain binding	3/105	0.012805487	KIDINS220/ERC1/SHISA6	3
GO:0017048	Rho GTPase binding	4/105	0.014042735	STXBP6/CORO1C/ARHGEF12/VAV3	4

GO:0015631	tubulin binding	6/105	0.018451388	TRAF3IP1/ARL8B/VAPB/DIXDC1/LYN/TRIM36	6
GO:0048487	beta-tubulin binding	2/105	0.018957779	ARL8B/VAPB	2
GO:1901981	phosphatidylinositol phosphate binding	4/105	0.019526335	SNX27/SNAP91/ZFYVE26/STXBP6	4
hsa05202	Transcriptional misregulation in cancer	5/43	0.002305109	SIX4/MEF2C/ZEB1/TFE3/ITGB7	5
hsa04810	Regulation of actin cytoskeleton	5/43	0.004895186	ARHGEF12/ACTN4/ITGB8/ITGB7/VAV3	5
hsa04512	ECM-receptor interaction	3/43	0.009088717	SV2C/ITGB8/ITGB7	3
hsa04510	Focal adhesion	4/43	0.016527038	ACTN4/ITGB8/ITGB7/VAV3	4
hsa04979	Cholesterol metabolism	2/43	0.025316333	VAPB/SORT1	2
hsa04514	Cell adhesion molecules	3/43	0.04035915	NRCAM/ITGB8/ITGB7	3
hsa04664	Fc epsilon RI signaling pathway	2/43	0.044101979	LYN/VAV3	2
hsa05211	Renal cell carcinoma	2/43	0.04526175	HIF1A/TFE3	2
